# P-1421. A socioeconomic index of Brazilian municipalities correlates with dengue cases severity status but not with lethality

**DOI:** 10.1093/ofid/ofae631.1596

**Published:** 2025-01-29

**Authors:** Guilherme Lira, Marcelo Takahashi, Victor Ota, Isabela Leitão

**Affiliations:** Universidade Federal do Rio de Janeiro, Rio de Janeiro, Rio de Janeiro, Brazil; Faculdade de Medicina FMUSP, Sao Paulo, Brazil, São Paulo, Sao Paulo, Brazil; Universidade Federal do Rio de Janeiro, Rio de Janeiro, Rio de Janeiro, Brazil; Universidade Federal do Rio de Janeiro, Rio de Janeiro, Rio de Janeiro, Brazil

## Abstract

**Background:**

Social determinants of health (SDOH) affect a wide range of health outcomes and risks. This includes differences in exposure and vulnerability to infectious diseases such as tuberculosis, COVID-19 and arboviral diseases. However, information on SDOH can be difficult to retrieve from medical records. We set out to explore how SDOH measured at populational level can affect natural history and lethality in dengue fever.

ORs from logistic regressions performed

**Figure:**
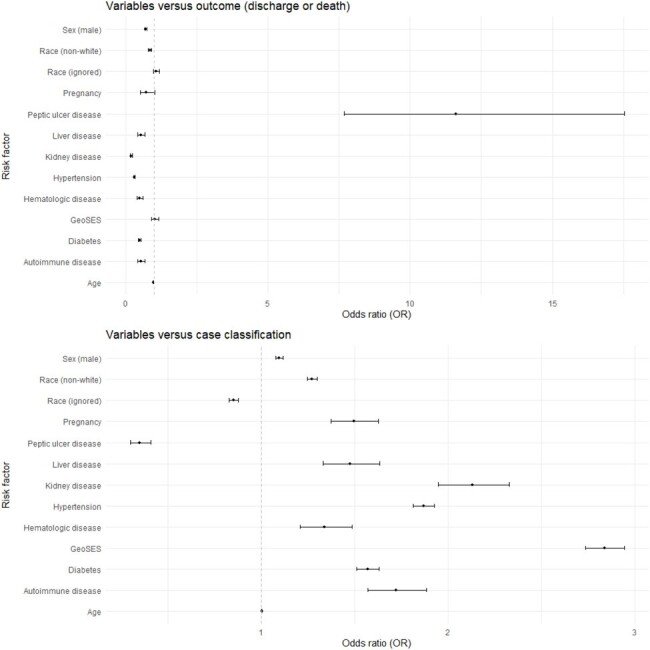

**Methods:**

We performed a retrospective, descriptive study of dengue fever cases in Brazil, using Ministry of Health openly available notification databases. We used GeoSES, a composite index that summarizes the main dimensions of the Brazilian socioeconomic context for research purposes, as a proxy for SDOH in given municipalities. Only laboratory confirmed cases were included. Outcomes considered were case classification (dengue without warning signs, dengue with warning signs or dengue with severity signs) and discharge or death by dengue. We performed logistic regressions of recognized risk factors alongside GeoSES as predictors for outcomes. R software version 4.3.1 was used for analysis. Code will be made public upon abstract acceptance.

**Results:**

We analyzed 2,137,462 cases notified between January 2014 and December 2023. In the proportional odds logistic regression model for case classification, the GeoSES index was associated with case classification (OR = 2.87, 95% CI 2.77-2.98; p-value < 0.0001), indicating an increased risk of dengue with warning signs or severity signs as the index increases. However, in the binomial logistic regression for discharge or death, the index showed no association (OR = 0.99, 95% CI 0.88-1.12; p-value = 0.98) with outcome, holding constant all other variables. Interestingly, peptic ulcer disease stood out as a protective factor against negative outcomes in dengue fever patients.

**Conclusion:**

As a seasonal disease with a recent upsurge in Brazil, reevaluating risk factors for dengue infection and complication is an ever-present necessity. Here, we demonstrate that the GeoSES index is not a strong predictor of dengue fever mortality. This does not excuse policymakers from improving structural conditions, but allows for rational evaluation in epidemic scenarioes.

**Disclosures:**

**All Authors**: No reported disclosures

